# New AI Technologies to Enrich Electronic Health Record Data Sets With Self-Report Scores in Geriatrics

**DOI:** 10.1093/geroni/igab046.1064

**Published:** 2021-12-17

**Authors:** Ricardo Pietrobon

**Affiliations:** SporeData, SporeData, North Carolina, United States

## Abstract

Although electronic health record data present a rich data source for health service researchers, for the most part, they lack self-report information. Although recent CMS projects have provided hospitals with incentives to collect patient-reported outcomes for select procedures, the process often leads to a substantial percentage of missing data, also being expensive as it requires the assistance of research coordinators. In this presentation, we will cover Artificial Intelligence-based based technologies to reduce the burden of data collection, allowing for its expansion across clinics and conditions. The technology involves the use of algorithms to predict self-report scores based on widely available claims data. Following previous work predicting frailty scores from existing variables, we expand its use with scores related to quality of life, i.e. mental health and physical function, and cognition. Accuracy metrics are presented both in cross-validation as well as external samples.

